# Determinants of frailty development and progression using a multidimensional frailty index: Evidence from the English Longitudinal Study of Ageing

**DOI:** 10.1371/journal.pone.0223799

**Published:** 2019-10-30

**Authors:** Nils Georg Niederstrasser, Nina Trivedy Rogers, Stephan Bandelow

**Affiliations:** 1 School of Applied Social Sciences, De Montfort University, Leicester, England, United Kingdom; 2 Department of Epidemiology and Public Health, University College London, London, England, United Kingdom; 3 Department of Physiology, Neuroscience and Behavioural Sciences, St. George’s University, St. George’s, Grenada; Nathan S Kline Institute, UNITED STATES

## Abstract

**Objective:**

To identify modifiable risk factors for development and progression of frailty in older adults living in England, as conceptualised by a multidimensional frailty index (FI).

**Methods:**

Data from participants aged 50 and over from the English Longitudinal Study of Ageing (ELSA) was used to examine potential determinants of frailty, using a 56-item FI comprised of self-reported health conditions, disabilities, cognitive function, hearing, eyesight, depressive symptoms and ability to carry out activities of daily living. Cox proportional hazards regression models were used to measure frailty development (n = 7420) and linear regression models to measure frailty progression over 12 years follow-up (n = 8780).

**Results:**

Increasing age (HR: 1.08 (CI: 1.08–1.09)), being in the lowest wealth quintile (HR: 1.79 (CI: 1.54–2.08)), lack of educational qualifications (HR: 1.19 (CI: 1.09–1.30)), obesity (HR: 1.33 (CI: 1.18–1.50) and a high waist-hip ratio (HR: 1.25 (CI: 1.13–1.38)), being a current or previous smoker (HR: 1.29 (CI: 1.18–1.41)), pain (HR: 1.39 (CI: 1.34–1.45)), sedentary behaviour (HR: 2.17 (CI: 1.76–2.78) and lower body strength (HR: 1.07 (CI: 1.06–1.08)), were all significant risk factors for frailty progression and incidence after simultaneous adjustment for all examined factors.

**Conclusion:**

The findings of this study suggest that there may be scope to reduce both frailty incidence and progression by trialling interventions aimed at reducing obesity and sedentary behaviour, increasing intensity of physical activity, and improving success of smoking cessation tools. Furthermore, improving educational outcomes and reducing poverty may also reduce inequalities in frailty.

## Introduction

Frailty is a common geriatric state [1] which affects roughly 10% of over 65 year olds [1] and is forecast to present extensive problems for health and social care systems across the globe because of rising life expectancy [2]. Frailty occurs as a consequence of age-related physiological decline in multiple organ systems and leaves individuals vulnerable to relatively minor stressors (e.g.: low/high temperatures; minor infections) that can lead to sudden and disproportionate changes in their health (i.e.: from a state of independence to dependence) [1]. To date there is no universally agreed definition of frailty [3]. Numerous conceptualisations of frailty have been used in studies, but few have been examined for reliability and validity [4]. There are two widely used frailty measures. The frailty phenotype [5] comprises of five highly specific physical variables that include unintentional weight loss, exhaustion, low physical activity, and slow gait speed. However many studies have not been able to follow the exact definition which makes comparisons between different studies difficult [6]. Notably there have been calls from researchers to include a cognitive domain within frailty constructs but even commonly used frailty constructs such as the frailty phenotype [5] have omitted cognition. The frailty index (FI) [7] defines frailty as a state and is measured by the accumulation of age-related health deficits which includes a wide range of health problems, including hearing, eyesight, cognitive problems and general health [7,8]. The conceptualisation of frailty is still evolving, but frailty defined using the deficit accumulation model which encompasses all known aspects of frailty (physical, affective, and cognitive) has been demonstrated to provide an adequate multidimensional representation of frailty [9–11]. Research on frailty has largely focused on physical conceptualisations of frailty [5,12] despite the strong link between cognitive decline and frailty development [13]. While cognitive decline and frailty are often considered as separate constructs there is evidence that they share common pathologies [14]. Older adults showing signs of frailty are at greater risk of adverse health outcomes including reduced functional independence [15], increased disability [16,17], poor quality of life [16], dementia [18], institutionalisation [19], and mortality [16,20]. Exploring modifiable risk factors that might delay the onset or reduce the progression of frailty is therefore important, especially in the context of an ageing society.

Studies investigating both the progression and incidence of frailty in the same populations are scarce, perhaps due to the lack of frailty follow-up data in studies. It is therefore uncertain whether the same risk factors affect progression as well as incidence of frailty. In this study we use the English Longitudinal Study of Ageing (ELSA) and make use of a 56-item multidimensional FI and follow-up of 12 years, covering 7 time-points to investigate risk factors for development and progression of frailty.

## Methods

### Sample

ELSA is a panel study that comprises multidisciplinary data from a representative sample of adults aged 50 years and over living in England. Full details on the ELSA sample and data collection are available elsewhere [21]. Participants were initially drawn from the Health Survey for England. Data have been collected over eight waves, with two-year intervals between waves. This study drew its sample from waves 2 (baseline) through 8, as previous waves did not contain data on potential frailty risk factors. Data for waves 2 (2004/2005) to wave 8 (2016/2017) were collected through self-completion questionnaires and nurse assessments. The London Multicentre Research Ethics Committee (MREC/01/2/91) granted ethical approval.

### Outcomes

#### Frailty index

A FI was created based on the procedure outlined by Searle et al. [7] and included disease-related symptoms, self-reported conditions, activities of daily living, mobility, cognition, chronic diseases, as well as self-rated health, vision, and hearing. All deficits were given a score of 0 to indicate no expression, 1 to indicate full expression and a score of between 0 and 1 for partial expression. The index was expressed as the number of deficits expressed divided by the number considered and had a range between 0 and 1. A score of 0.25 or lower [22] indicates the absence of frailty, while higher scores indicate frailty. The FI is comparable across studies, even when different numbers (> 30 deficits) or types of deficits are counted, as shown by a multitude of studies [11,22–27].

#### Candidate variables

Based on the literature, candidate variables were selected as potential predictors of frailty. The variables did not form part of the FI and included: *sex*, *age*, *pain*, *physical activity level*, *wealth quintiles*, *educational qualifications*, *smoking*, *lower body strength*, *social isolation*, *loneliness and BMI and waist-hip ratio* as indicators of *obesity*. See appendix for a detailed description of the candidate variables.

### Statistical approach

Missing data were imputed using the R-package missFOREST [28]. Descriptive statistics were computed on sample characteristics and questionnaire scores using the imputed data set. All analyses were performed using R version 3.2. To explore the relationship between the predictors and the FI, partial correlations, correcting for participants’ age, were computed. The linear regression analysis was conducted on the entire data set (n = 8780) to measure frailty progression, while the Cox proportional hazards regression excluded those already classified as frail (FI > 0.25; n = 7420; events = 2441) at baseline.

For the Cox proportional hazards regression, FIs from waves 2 through 8 were combined in a data set with baseline predictors. Wave was used to denote survival time until development of frailty. Frailty development was operationalised by dichotomising the FI, with values ≥ 0.25 indicating frailty and < 0.25 indicating absence of frailty [22]. The R-package “survival” was used to compute the proportional hazards regression and “survminer” for visualising the results [29]. Univariate proportional hazards regression analyses were used to determine which predictors were added to the final multivariate model. To rule out potential reverse causation, sensitivity analyses were carried out, excluding participants who were defined as frail at baseline (wave 2) and those who became frail in wave 3.

Prior to the regression analyses, correlational analyses (spearman) were conducted to examine the bivariate relationships among the predictor variables and the FI. The baseline FI was entered into the regression equation as a covariate in step 1. All candidate variables were entered in the regression equation during step 2 (except social isolation, which was not correlated with the FI). Diagnostic tests of tolerance and variance inflation revealed that all of the measures fell within acceptable ranges of collinearity (variance inflation factors < 4).

Reference categories and units for the candidate variables were as follows: *Pain* “no, mild, moderate, or severe pain”), *Physical activity level* (sedentary (reference category), mild, moderate, and vigorous), *Wealth* (quintiles; lowest quintile is reference category), *Smoking* (current or previous smoker (reference category) vs. abstinence), *Lower body strength* (time to perform five chair rises; higher values indicated poorer strength), *Sex* (male (reference category) vs. female), *Age*, *general obesity* (Body Mass Index (BMI): measured as “kg/m^2^” and defined as underweight (BMI < 18.5), normal weight (reference category; BMI between 18.5 and 25), overweight (BMI between 25 and 30), and obese (BMI > 30), *Social isolation* (as described in [30], higher values indicated greater social isolation), *Loneliness* (Revised UCLA Loneliness Scale [31], higher values indicated greater loneliness), *abdominal obesity* (Waist-hip ratios exceeding 0.90 for men and 0.85 for women were counted as obesity [32]; normal weight was the reference category), *and educational qualifications* (no educational qualification (reference category) vs. any educational qualification).

## Results

### Sample characteristics

Baseline (wave 2) comprised of data from 8780 individuals (mean age 66.93, *SD* = 10.08; 3949 males, 44.98%). Table 1 presents the means and standard deviations as well as counts for the FIs and candidate variables at baseline for the complete dataset. Table 2 shows the partial correlations between the FI and the candidate variables that were measured on either interval or ratio scale corrected for participants’ age. The partial correlations, accounting for differences in participants’ ages, revealed that social isolation did not correlate with lower body strength.

**Table 1:** Sample overview.

Variables	
Frailty Index Wave 2 - mean (SD)	0.13 (0.11)
Wave 3 - mean (SD)	0.14 (0.12)
Wave 4 - mean (SD)	0.16 (0.12)
Wave 5 - mean (SD)	0.17 (0.13)
Wave 6 - mean (SD)	0.19 (0.14)
Wave 7 - mean (SD)	0.20 (0.15)
Wave 8 - mean (SD)	0.22 (0.16)
Waist-hip ratio (n)	
**Normal waist-hip ratio**	2962 (33.74%)
High waist-hip ratio	5818 (66.26%)
Loneliness (SD)	4.18 (1.44)
Pain intensity - mean (SD)	1.73 (1.03)
Social Isolation–mean (SD)	2.33 (1.29)
Wealth (quintiles; n)	
**Poorest**	1601 (18.23%)
	1749 (19.92%)
	1756 (20.00%)
	1791 (20.40%)
Richest	1883 (21.45%)
Smoking history (n)	
**Yes**	5587 (63.63%)
No	3193 (36.37%)
Chair Rises time (for 5 rises) - mean (SD)	12.66 (4.70)
Sex (n)	
**Male**	3949 (44.98%)
Female	4831 (55.02%)
Age - mean (SD)	66.93 (10.08)
Education (n)	
**No educational qualification**	3426 (39.02%)
Any educational qualification	5354 (60.98%)
Physical activity level (n)	
**Sedentary**	552 (6.29%)
Mild	2247 (25.59%)
Moderate	4393 (50.03%)
Vigorous	1588 (17.74%)
BMI categories (n)	
Underweight	62 (0.71%)
**Normal**	1969 (22.43%)
Overweight	4370 (49.77%)
Obese	2379 (27.10%)

Notes: N = 8780; unless otherwise indicated, variables refer to those taken at wave 2; for categorical variables reference categories are printed in bold;

**Table 2:** Partial relationships (corrected for participants’ age).

	Chair Rises time	Pain Intensity	Social Isolation	Loneliness
Frailty Index Wave 8	0.49**	0.44**	-0.02*	0.38**
Chair Raises time		0.28**	-0.01	0.23**
Pain Intensity			0.03*	0.20**
Social Isolation				0.07**

Note: N = 8780;

* *p* < .05;

** *p* < .01;

### Development of frailty

In unadjusted models, social isolation did not influence frailty development and was therefore omitted from further models. Next, we fitted a multivariate Cox hazards ratio analysis using the significant univariate predictor variables to describe how the factors jointly impacted on the incidence of frailty. Visual inspection of each covariate’s scaled Schoenfeld residuals plotted against survival time supported the assumption of proportional hazards.

**Fig 1:**
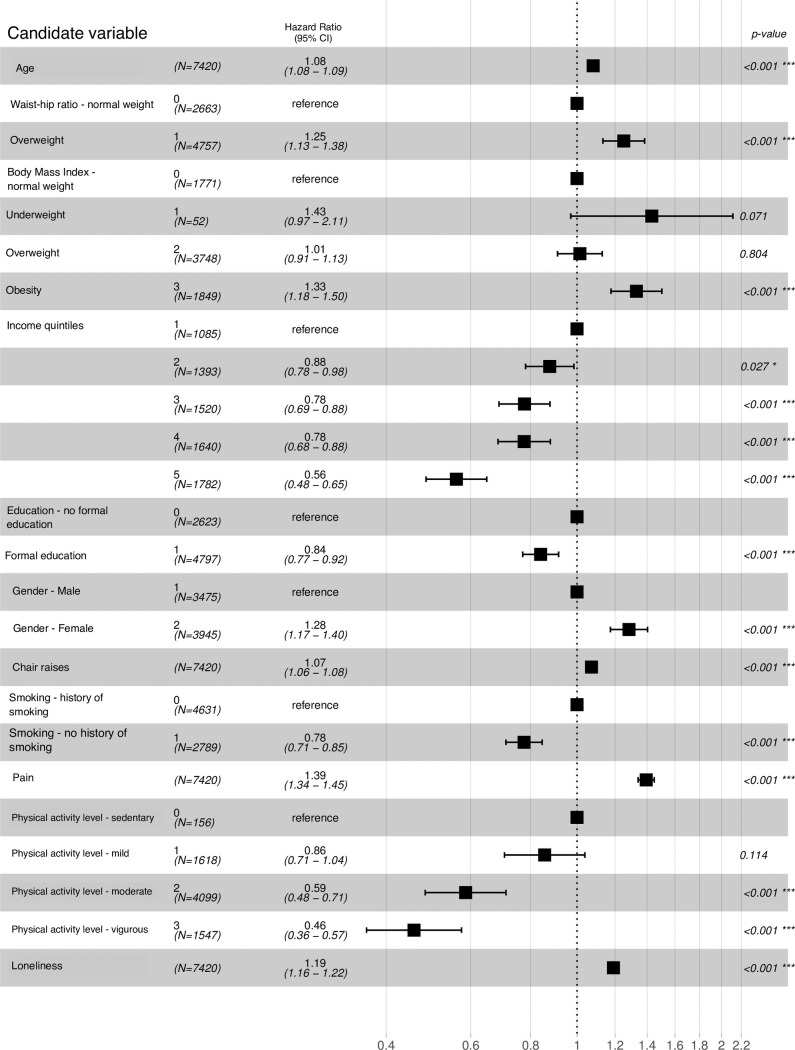
Hazard ratios for frailty development. ***values are rounded.

A total of 7240 non-frail participants were included in the analysis, of which 2441 developed frailty over the course of 12 years (Fig 1). Age was associated with quicker development of frailty (HR = 1.08, CI = 1.08–1.09). Compared to those with a BMI in the normal range, participants classed as obese (HR = 1.33, CI = 1.18–1.50) had a higher risk of becoming frail. Participants with a high waist-hip ratio had a 1.25-increased risk (CI = 1.13–1.38) of becoming frail compared to those with healthy ratios. Higher wealth was associated with lower frailty incidence (e.g. 5^th^ quintile HR = 0.56, CI = 0.48–0.65). Any education compared to no education showed a protective effect against early development of frailty (HR = 0.84, CI = 0.77–0.92). Compared to males, females were more likely to become frail (HR = 1.28, CI = 1.17–1.40). Poor lower body strength was identified as being associated with higher frailty incidence (HR = 1.07, CI = 1.06–1.08). Abstinence from tobacco (HR = 0.78, CI = 0.71–0.85) was associated with slower development of frailty, while higher levels of pain intensity (HR = 1.39, CI = 1.34–1.45) and loneliness (HR = 1.19, CI = 1.16–1.22) were associated with higher risk of developing frailty. Compared to sedentary individuals, those engaging in moderate (HR = 0.59, CI = 0.48–0.71) or vigorous physical activity (HR = 0.46, CI = 0.36–0.57) were less likely to become frail. These results show that a person with an average age of 67, who takes part in mild physical activity or is sedentary and is a current or previous smoker has a 59% chance of becoming frail by the time they are roughly 79 years old. In contrast, a person of the same age, who takes part in moderate or vigorous physical activity and has never smoked has a 22% chance of becoming frail over the same period. Similarly, a 67-year-old individual who is overweight or obese and smokes or has a smoking history has a 37% chance of becoming frail, whereas a person with a healthy weight that has never smoked has a 19% chance of developing frailty.

In the sensitivity analysis, participants were excluded if they became frail in the 24 months following baseline measurements, but this had a negligible effect on the associations between the potential frailty determinants and frailty incidence.

#### Frailty progression

Spearman correlation coefficients between the FI and the candidate variables that were measured on either interval or ratio scale were calculated to determine their inclusion into the regression analysis.

#### Linear regression analysis

As shown in Table 3, baseline frailty levels contributed significantly to the prediction of frailty levels at wave 8, explaining 56% of the variance. Addition of the candidate variables in the final step of the analysis yielded a 19% increase in explained variance in frailty at wave 8. Beta weights for the final regression equation indicated that frailty at baseline (β = .45, *p* < .01), age (β = 0.36, *p* < .01), pain intensity (β = .03, *p* < .01), lower body strength (β = .13, *p* < .01), and loneliness (β = 0.04, *p* < .01) contributed significant unique variance to the prediction of frailty at wave 8. Differences in frailty at wave 8 were found for several variables. Individuals with a high waist-hip ratio reported greater frailty at wave 8 compared to those with normal waist-hip ratio (β = .04, *p* < .01). Similarly, participants whose BMI was classed as obese (β = .02, *p* < .01) were significantly more frail at wave 8 compared to participants with BMI in the healthy range. Overweight and underweight as indicated by BMI were not predictors of frailty (overweight: β = -.01, *p* = .15; underweight: β = .01, *p* = .24). Participants in higher wealth quintiles were significantly less frail compared to those in the lowest quintile (2^nd^ quintile: β = -.03, *p* < .01, 3^rd^ quintile: β = -.05, *p* < .01, 4^th^ quintile: β = -.05, *p* < .01, 5^th^ quintile: β = -.08, *p* < .01). Participants with any completed formal education at baseline had lower levels of frailty 12 years later (β = -.03, *p* < .01). Furthermore, male participants were less frail than female participants at wave 8 (β = .02, *p* < .01) even after taking account of baseline frailty. Abstinence from tobacco smoking at baseline was associated with less frailty 12 years later (β = -.03, *p* < .01). Moderate (β = -.07, *p* < .01) or vigorous (β = -.07, *p* < .01) physical activity was associated with less frailty compared to a sedentary lifestyle.

**Table 3:** Regression analysis predicting frailty at Wave 8.

Variables	β	R^2^ change	F-change	*p* value
Step 1		0.56	11224 (1, 8778)	< .01
Frailty wave 2	0.45**			
Step 2		0.19	373.05 (18, 8760)	< .01
Waist-hip ratio	0.04**			
BMI - underweight	0.01			
BMI - overweight	-0.01			
BMI - obese	0.02**			
Wealth– 2^nd^ quintile	-0.03**			
3^rd^ quintile	-0.05**			
4^th^ quintile	-0.05**			
5^th^ quintile	-0.08**			
Education (none vs. formal)	-0.03**			
Female gender	0.02**			
Age	0.36**			
Chair raises in sec.	0.13**			
Smoking history (yes vs. no)	-0.03**			
Pain intensity	0.03**			
PA - mild	-0.00			
PA - moderate	-0.07**			
PA - vigorous	-0.07**			
Loneliness	0.04**			

Note: N = 8780;

* *p* < .05;

** *p* < .01; Values in parentheses are degrees of freedom. Beta weights are from the final regression equation;

## Discussion

In this study, we found that higher baseline frailty score, increasing age, low wealth, low levels of education, obesity, high waist-hip ratio, being female, lower body strength, being a smoker or having a history of smoking, pain, low intensity of physical activity or sedentary behaviour, and loneliness were predictors of both frailty progression and development. Social isolation was not a predictor of frailty development and progression.

The strengths of the study included the use of a large representative sample of older adults living in England, use of a validated multidimensional FI, a range of objective and self-reported predictor variables and a follow-up period of up to 12 years. Caution must be used when interpreting the study findings. Given the study’s longitudinal nature it is possible that the frailest participants died or were lost to follow-up between assessment points. To counteract this survivor bias, data were imputed for all participants taking part from wave 2 onwards. Nevertheless, data imputation methods, while sophisticated, are not capable to perfectly reproduce missing data. The current FI did not contain deficits pertaining to social frailty [33], such as perceived social isolation or loneliness (considered as exposures in this study), although these have previously been recommended for inclusion in a multidimensional frailty index [34]. The identified risk factors explain significant chunks of variance above what is explained by existing symptomology and therefore make a meaningful contribution to the outcome predictions; however residual confounding cannot be excluded.

Age was the strongest predictor of frailty, corroborating previous research demonstrating a strong positive relationship between age and frailty [35–37]. Nevertheless, other variables including modifiable risk factors (e.g.: obesity and sedentary lifestyle) were independently associated with frailty development and progression. Obesity and abdominal (waist-hip ratio) obesity but not overweight predicted greater progression and higher risk of becoming frail [38,39]. These findings are in line with previous studies on midlife physical functioning demonstrating that it is the magnitude of adiposity that is of prime importance, with highest levels of BMI being particularly deleterious to physical health [40]. Lipid depositions and lipid infiltration in muscle fibres may contribute to frailty by reducing mobility and promoting the loss of muscle strength [41]. Furthermore, excess body fat puts individuals at risk of developing proinflammatory [42] and prothrombotic states [43], as well as vascular events and hyperinsulinemia [44]. The independent contribution of BMI and abdominal obesity on frailty progression and incidence may be rooted in waist-hip ratio better reflecting body fat deposits, compared to BMI. Greater BMI does not necessarily reflect poor health, because it does not distinguish between fat mass and muscle mass. Furthermore, BMI categories used to determine obesity have been challenged by previous investigations, suggesting different cut-offs depending on age [45]. A state of obesity may also lead to more joint wear and tear and reduced physical activity [46]. Sedentary levels of physical activity predicted frailty after 12 years and quicker progression compared to moderate or vigorous physical activity, corroborating findings from previous research [47]. Physical activity may improve physical function, promote healthy weight, bone mass, muscle function, prevent falls, as well as improve general health [48], thereby slowing the onset and progression of frailty. Increasing physical activity can have beneficial effects on obesity, stress, loneliness in the case of group exercises, and muscular strength, which have been suggested as potential risk factors of increased frailty [49].

Low muscular strength was associated with increased frailty and quicker development of frailty, consistent with existing findings suggesting a negative association between muscular strength and frailty [50–52]. Low physical strength in advanced age is often the result of sarcopenia, which is the age-related loss of muscle mass [53]. Sarcopenia is essential in the pathogenesis of frailty and renders individuals at greater risk for adverse health effects, such as falls; however there is evidence that exercise interventions may help to slow or reverse sarcopenia and resultant strength loss [54].

Non-smokers were less likely to become frail or have severe frailty compared to current or previous smokers, confirming previous research showing that smoking is detrimental for frailty [55–57]. Smoking is associated with an increased risk of developing numerous diseases, including arterial vascular disease [58], obstructive pulmonary disease (COPD) [59], stroke [60], and coronary heart disease [61]. Furthermore, these debilitating diseases have a knock-on negative effect on physical, psychological and social health which all contribute to frailty [57].

High levels of pain were a significant risk factor for frailty. We are aware of only one study that has examined the links between pain and physical frailty [62] but it has been suggested that pain may predispose individuals to lead a sedentary lifestyle, as individuals’ inclination to move is reduced, due to fear of experiencing pain [63]. Pain contributes to deteriorations in physical function and capacity [64], which are integral contributors to frailty. Furthermore, chronic pain is associated with increased levels of depression [65] and anxiety [66], which may affect psychosocial aspects of frailty.

Loneliness was a significant predictor of incidence and progression of frailty, corroborating previous findings [67,68]. On the other hand, social isolation was not associated with the progression or development of frailty, confirming that loneliness and social isolation are distinct states [69]. Further, it implies that social isolation must not necessarily be interpreted as a negative state, as there is variation in the amount of social inclusion individuals seek (i.e.: socially isolated individuals may not be unhappy). Social isolation was quantified as the frequency of contacts and thus did not contain an affective component. Perceived social isolation, which does contain an affective component, might be a better representation of individuals’ social isolation. Furthermore, the current FI did not include social aspects of frailty [33,34], which may explain why social isolation did not predict frailty. It remains elusive whether loneliness is a result of increased frailty or a causal antecedent. The current study, due to its longitudinal nature, suggests that loneliness precedes frailty. Nevertheless, intervention studies aimed at reducing loneliness are warranted to confirm a causal link.

Education and wealth emerged as strong non-modifiable risk factors for quicker development of frailty, suggesting a link between lower wealth and frailty [70,71] and highlighting the importance of making available health care and education for all strata of society. Negative health outcomes and behaviours have been linked with lower wealth, such as low use of preventive care [72], which may contribute to frailty, especially at older age when individuals are more vulnerable to stressors [73,74]. Previous investigations have suggested that higher education protects against cognitive decline [75], but less so physical frailty [76], when other factors were taken into account. The protective effect of educational attainment lends credence to the cognitive reserve hypothesis [77] and the link between lower wealth, low educational attainment and negative health outcomes [78]. It further highlights the importance of including cognitive aspects in a multidimensional concept of frailty.

Consistent with other studies, this study shows that females are more likely to be frail than males, suggesting that male gender is a protective factor when it comes to development and progression of frailty. Difference in muscle mass, physical activity, higher fat percentages, and widowed status may account for gender differences in frailty [79–81]. Intervention studies are needed to understand the pathways that lead to a gender effect on frailty status in older adults.

In conclusion, this study shows a range of modifiable social and behavioural risk factors are important for the development and progression of frailty. Cessation of smoking, promotion of physical activity and weight loss in obese adults may be beneficial for preventing frailty in older adults. Recent evidence suggests that multi-modal interventions that offer multiple health-promoting components such as cognitive stimulation, stress reduction, reduction in sedentary behaviour and loneliness are associated with improved frailty outcomes [82]. Carefully designed intervention studies are required to understand the most cost-effective solutions to protect against frailty development and progression.

## Appendix

### Pain

Participants were asked to rate the intensity of the pain they perceive most of the time, ranging from “mild” (1) to “severe” (3). Participants who indicated not experiencing recurrent or frequent pain were recorded as scoring “no pain” (0).

### Physical activity level

To determine physical activity levels, participants were shown a series of prompt cards depicting activities of various intensities, e.g. vigorous (swimming or tennis), moderate (gardening or washing the car), and mild (laundry). These prompt cards were designed to aid the process of participants indicating how often they engage in that type of physical activity in their leisure time. Participants had the following response options: more than once per week, once per week, one to three times per month, and hardly ever. Participants were then divided into four categories of physical activity level (sedentary, mild, moderate, and vigorous), based on the highest intensity activity they perform at least once per week.

### Wealth quintiles

Wealth was determined by dividing participants into quintiles based on their net wealth. Net wealth was quantified as the net sums of housing wealth, physical wealth (including additional property wealth, wealth related to business and other physical assets) and financial wealth.

### Smoking

Participants were asked regarding their current cigarette smoking habits and whether they had a history of cigarette smoking.

### Lower body strength

Chair rises were used as a measure of lower body strength. A nurse instructed participants to stand up from a chair without the use of their arms, as quickly as possible five or ten times, depending on age. Instructions specified that participants aged 70 and over attempt five rises, while those younger than 70 attempt ten rises. The time it took participants to perform these rises was recorded. For participants who performed ten rises, the time it took to perform five rises was noted also. Therefore, the time it took it took all participants to complete five rises was included in the analysis. Consequently, lower values on this variable represent greater lower body strength.

### Sex

Sex was self-reported by participants during interviews.

### Age

Ages over 90 were collapsed into a single age group, as to protect participants’ identities.

### Body mass index (BMI)

BMI (kg/m^2^) was calculated by dividing body weight (kg) by standing height (meters) squared. Weight and height were measured by a trained nurse. The scales used during the nurse visits had a maximum weight capacity of 130 kg and so participants whose weight exceeded 130 kg could not be measured. Participants were categorised as underweight (BMI < 18.5), normal weight (BMI between 18.5 and 25), overweight (BMI between 25 and 30), and obese (BMI > 30).

### Social isolation

As described in [30], social isolation was derived as follows: being unmarried or not living with a partner (scored as 1), less than monthly contact with other family, friends and children (each scored as 1), and non-participation in any social activities (scored as 1). Resultant scores ranged between 0 and 5, with higher scores indicating greater social isolation.

### Loneliness

Loneliness was assessed using the Revised UCLA Loneliness Scale [31]. This 3-item instrument yielded scores between 3 and 9, whereby higher scores indicated greater loneliness.

### Waist-hip ratio

The ratio between hip and waist circumference was reported as an indication of abdominal obesity. Waist-hip ratios exceeding 0.90 for men and 0.85 for women were counted as obesity [32]. A trained nurse measured participants’ waist and hip circumferences twice, to ensure precision and the mean of both measurements was reported in centimetres and the ratio calculated.

### Education

This dichotomous variable was coded as “1” for any educational attainment by the end of the respective wave and “0” to signify no formal education.
